# Establishing a Multidisciplinary Framework to Study Drug‐Drug Interactions of Hormonal Contraceptives: An Invitation to Collaborate

**DOI:** 10.1002/psp4.12357

**Published:** 2018-10-23

**Authors:** Lawrence J. Lesko, Valvanera Vozmediano, Joshua D. Brown, Almut Winterstein, Ping Zhao, Jörg Lippert, Joachim Höchel, Ayyappa Chaturvedula, Annesha White, Stephan Schmidt

**Affiliations:** ^1^ Center for Pharmacometrics and Systems Pharmacology Department of Pharmaceutics College of Pharmacy University of Florida Lake Nona (Orlando) Florida USA; ^2^ Department of Pharmaceutical Outcomes and Policy College of Pharmacy University of Florida Gainesville Florida USA; ^3^ The Bill & Melinda Gates Foundation Seattle Washington USA; ^4^ Clinical Pharmacometrics Bayer AG Pharmaceuticals Wuppertal Germany; ^5^ Bayer AG Berlin Germany; ^6^ Department of Pharmacotherapy University of North Texas Health Science Center Fort Worth Texas USA

## Abstract

Hormonal contraceptive agents (HCAs) are widely used throughout the world, and women taking HCAs are likely to take other medications. However, little is known about the clinical effect of most drug‐drug interactions (DDIs) associated with HCAs. A team of interdisciplinary outcomes and pharmacometric researchers from academia and industry jointly engage in a research project to (i) quantitatively elucidate DDI impacts on unintended pregnancies and breakthrough bleeding, and (ii) establish a DDI‐prediction framework to inform optimal use of HCAs.

Hormonal contraceptive agents (HCAs) are widely used throughout the world, mainly to prevent unintended pregnancies. In its 2015 report “Trends in Contraceptive Use Worldwide” the United Nations state that “64% of married or in‐union women of reproductive age worldwide were using some form of contraception.” This percentage is typically much lower in the least developed countries (~40%), particularly in Africa (33%), compared with the other major geographic areas ranging from 59% in Oceania to 75% in Northern America. Nearly 800 million married or in‐union women are projected to be using contraception in 2030 and growth in the number of contraceptive users is expected to be uneven across regions.^1^

In the United States, about half of all pregnancies are unintended at conception and about half of these occur among users of a contraceptive method.^2^ The most recently approved HCAs have perfect‐use failure rates of about 1–2% estimated in phase III clinical trials, whereas the estimated failure rates of the typical use in the general population are closer to 9%.^3^ There are several factors that can contribute to an increase in failure rates during postmarketing use, including noncompliance with the regimen, but also polypharmacy with potential for drug‐drug interactions (DDIs), which may result in a clinically meaningful increase of unintended pregnancies or breakthrough bleedings. Societal cost savings from pregnancies averted by proper dose adjustment of HCAs has been estimated to be as much as $5,431 in medical, welfare, and social services costs per woman and child from conception up to 2 years after a pregnancy.^4^

For example, some anti‐epileptic drugs, such as carbamazepine, felbamate, or phenytoin (perpetrators), may induce cytochrome P450 (CYP)3A4 and, hence, increase the clearance of HCAs (victims). Therefore, women with epilepsy taking HCAs are at particular risk for DDIs.^5^, ^6^ When used properly, HCA failure is 1% in healthy women but it is 3–6% in women with epilepsy.^6^ Although, in the past, studies and recommendations on the interaction between HCAs and anti‐epileptic drugs have often focused on the alteration of the estrogen component of the agents, it has now become clear that it is, in fact, the progestin component that provides the major part of the contraceptive effect of modern combined oral contraceptives.^6^ In addition to CYP‐mediated DDIs, the relevance of other major metabolic enzymes, such as sulfotransferases or glucuronosyltransferases for ethinyl estradiol, for DDIs needs to be better understood.

Mechanistic understanding of DDIs with HCAs is important when attempting to determine the impact of drug‐induced changes in progestin and estrogen exposure on unintended pregnancies and breakthrough bleeding. Such understanding is hampered by the fact that many of the HCAs were developed > 50 years ago with different standards of clinical trial development compared to today, so that knowledge gaps on the metabolic pathways of progestins and ethinyl estradiol remain, albeit this could be tackled in part by conducting *in vitro* experiments. As a consequence, our knowledge on the potential for pharmacokinetic (PK; or pharmacodynamic (PD)) DDIs with HCAs is incomplete.^6^ The use of model‐informed drug development and discovery approaches including physiologically based pharmacokinetic (PBPK) and model‐based meta‐analysis (MBMA) can offer integrated assessment to bridge these gaps. If unwanted pregnancies can be averted by prescribers having adequate information about HCA DDIs, the approximate cost savings can be over $1 billion annually.^4^

The importance of DDIs for HCAs has not gone unnoticed by the US Food and Drug Administration (FDA). A workshop held in November 2015 served as a landscape analysis of the issue and identified unmet needs in this area. Subsequently, the FDA published a draft guidance for industry on labeling for combined hormonal contraceptives focused, in part, on DDIs (Section 7 of Guidance^7^).

Ultimate understanding of clinical implication of HCA DDIs requires the integrated use of different quantitative and systems pharmacology approaches and outcomes research (introduced in the next section), which can reliably query real‐world outcomes data for evidence on unintended pregnancies and breakthrough bleeding followed by an evaluation of their mechanistic plausibility. The integrated approach, complemented by the use of dose‐exposure‐response analyses, can be used to support dosing or treatment recommendation associated with HCA DDIs in drug labels.

## Integration and collaboration

**Figure 1:**
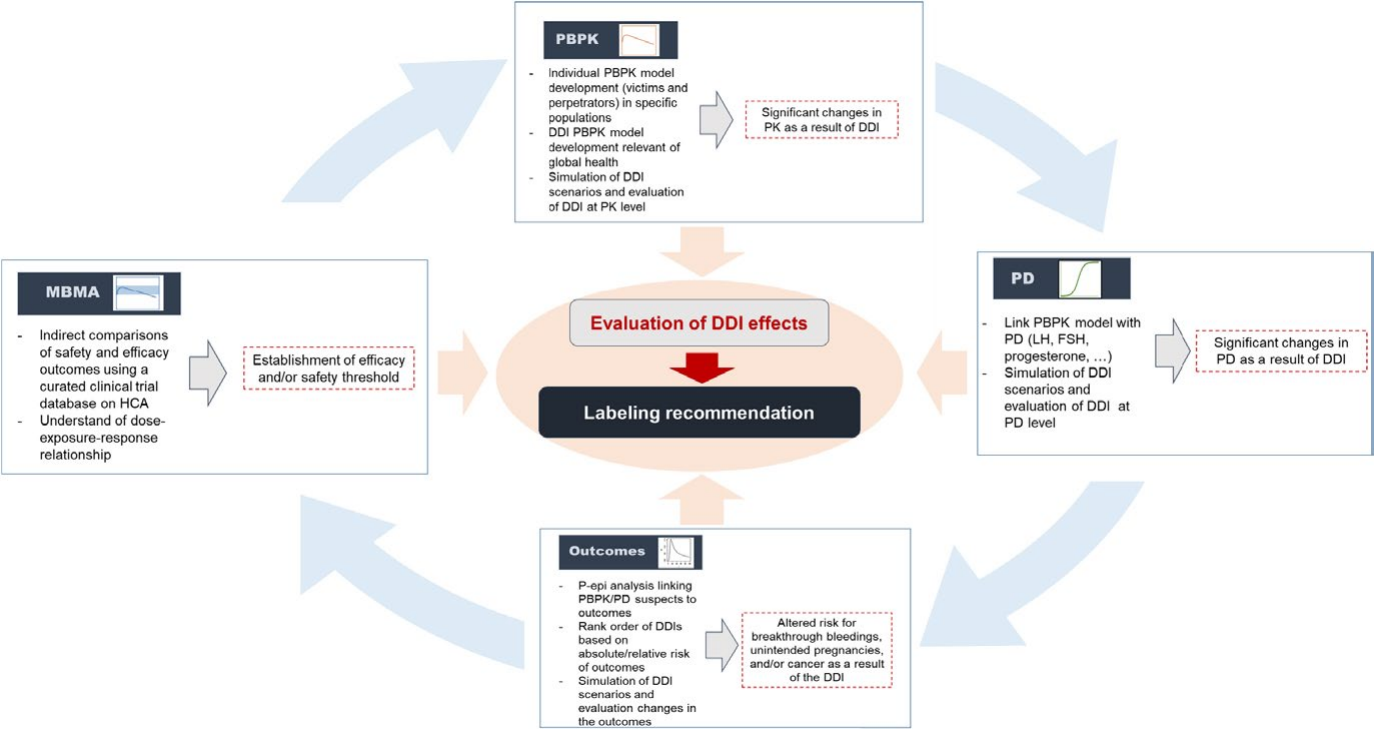
Key steps in the integrative pharmacometric model‐based assessment on drug‐drug interactions (DDIs) with hormonal contraceptive agents. FSH, follicle stimulating hormone; HCA, hormonal contraceptive agent; LH, luteinizing hormone; MBMA, model‐based meta‐analysis; PBPK, physiologically based pharmacokinetic; PD, pharmacodynamic; P‐epi, pharmacoepidemiologic; PK, pharmacokinetic.

The Center for Pharmacometrics and Systems Pharmacology at the University of Florida and the Bill & Melinda Gates Foundation have agreed to collaborate on studying the impact of DDIs on unintended pregnancies as well as on breakthrough bleeding. The proposed research strategy rests on the integration of exposure data from physiologically based DDI models, dose‐response relationships derived from MBMA, and real‐world outcomes data (**Figure** **1**) as well as interdisciplinary and interinstitutional collaboration among academia, industry, and foundations.

The first component in enabling this integrated research strategy, which serves for signal detection, is the assessment of real‐world medication and pregnancy outcomes data. It uses large administrative claims databases, including the Truven MarketScan databases (~160 million commercially insured) and the Medicaid Analytic Extract data (“MAX,” 29 US states of ~60 million Medicaid insured). Each database includes longitudinal records of medical and pharmacy utilization of women with date‐stamped records allowing for near exact assessment of utilization of medications and the occurrence of relevant outcomes. Combined with active comparator study designs, propensity score methodology, and regression modeling, these analyses will assess the absolute and relative increased risk of outcomes across a number of potential CYP interactions for both acute and chronic medications.

The second component of this integrated research strategy is to determine if real‐world outcomes can be explained by changes in HCA (victim drug) exposure due to PK DDIs. Perpetrator drugs (CYP inducers; e.g., rifampin, carbamazepine, and phenytoin or inhibitors, e.g., itraconazole fluconazole, indinavir, and boceprevir) will be selected in accordance with the FDA's guidance on labeling HCAs and will be used in the mechanistic evaluation of DDI through PBPK modeling and simulation approach. The appeal of PBPK for this research is that it is a mechanistic strategy that provides quantitative insights to complement empirical or “static” strategies upon which to explore and understand the mechanistic rationale critical for enabling data‐driven selection of doses for combined estrogen‐progestin products and/or single progestin products. In this regard, colleagues from Bayer AG have agreed to share the information on several in‐house HCA‐DDI studies to help in the development of our PBPK platform. Bayer has been working as part of a network of public and private partners to enable women and girls to assert their right to self‐determined family planning worldwide for 50 years. In recognition of this long‐standing commitment, Bayer was accepted in 2007 as a member of the Reproductive Health Supplies Coalition – as the first pharmaceutical company. Bayer supports family‐planning programs in over 130 countries providing access to modern contraceptives for women. Bayer is doing this in joint projects with the United States Agency for International Development and nongovernmental organizations, such as the United Nations Population Fund. Within these programs, Bayer offers a broad range of hormonal contraception methods, like oral contraceptives, injections, and implants. In this project, Bayer will compile, de‐identify, and provide proprietary HCA DDI study data (in line with patient informed consent and European and German data privacy legislation). Data will be made available via www.open-systems-pharmacology.org, an open source platform initiated by Bayer in 2017 to further develop its formerly commercial PBPK platform PK‐Sim. The PBPK models are initially developed for HCAs administered orally and then further expanded to other administration routes (i.e., injectable and intra‐uterine devices) to stepwise evaluate the complex interaction among tissue, delivery system, and formulation of HCA drug products.

The third component of the integrated research strategy is to derive dose‐response relationships of the target HCAs via an MBMA. The MBMA utilizes PD models, such as maximum effect (E_max_) models to understand dose response relationships from summary level clinical trial outcomes data. It is a useful approach to make indirect comparisons of safety and efficacy outcomes, where randomized controlled trials with head‐to‐head comparisons are not available. Assuming treatment effects are exchangeable between randomized controlled trials, a mixed effects model for k‐outcomes will be fit to the summary level data using the clinical trial outcomes data database using unintended pregnancies and breakthrough bleeding as respective efficacy and safety end points. This MBMA will be linked to PBPK models to derive exposure thresholds (e.g., minimum effective concentration) for estrogen and progestins that can serve as PK surrogate when evaluating the clinical meaningfulness of HCA DDIs.

In summary, the first component will serve as the basis to identified clinically relevant DDIs based on pharmacoepidemiologic analyses of DDIs in real‐world populations. The second component will establish the mechanistic basis of identified DDIs using physiologically based DDI models. Respective changes in HCA exposure will then be linked to response data derived from an MBMA in order to identify efficacious and safe exposure thresholds of HCAs, which may also be directly used in the evaluation of the DDI outcomes and to support dosing recommendations and package inserts. Ideally, the outcome of this analysis will be qualified by data from prospectively designed clinical trials for HCAs.

## Invitation to collaborate

Finally, a major goal of this collaboration will be to ensure open access to a model‐based PBPK platform containing system and drug databases. This platform will serve as a cutting‐edge repository, where participating scientists and clinicians can exchange, revise, and apply information and tools with the common aim of ongoing improvement in the evaluation of HCA DDIs and potentially benefit from DDI clinical study waivers from regulatory agencies. Partners of this project welcome involvement of other organizations that wish to contribute with data (e.g., University of Washington database), ideas, modeling technology, or expertise (e.g., Simcyp or GastroPlus) to this open access model of HCA DDIs. We expect that this Bill & Melinda Gates foundation‐sponsored project illustrates how cooperation between diverse organizations and disciplines are key for leveraging noncompeting interests to improve the use of HCAs for patients globally. The open source nature of this effort in conjunction with future publication of model details (e.g., detailed parameterization and parameter values) will allow easy uptake for researchers and physician scientists, including those using different tools, around the world.

## Funding

This work was supported by the Bill & Melinda Gates Foundation.

## Conflict of Interest

As an Associate Editor for *CPT: Pharmacometrics & Systems Pharmacology*, Ping Zhao was not involved in the review or decision process for this paper. Lawrence J. Lesko, Valvanera Vozmediano, Joshua D. Brown, Almut Winterstein, and Stephan Schmidt are employees of the University of Florida, Ayyappa Chaturvedula and Annesha White are employees of the University of North Texas Health Science Center, Jörg Lippert and Joachim Höchel are employees of Bayer AG Pharmaceuticals, and Ping Zhao is a full‐time employee of the Bill & Melinda Gates Foundation.
